# Crosstalk Between Mast Cells and Adipocytes in Physiologic and Pathologic Conditions

**DOI:** 10.1007/s12016-020-08785-7

**Published:** 2020-03-25

**Authors:** Daniel Elieh Ali Komi, Farzaneh Shafaghat, Mark Christian

**Affiliations:** 1 Immunology Research Center, Tabriz University of Medical Sciences, Tabriz, Iran; 2 Department of Immunology, Tabriz University of Medical Sciences, Tabriz, Iran; 3 School of Science and Technology, Nottingham, NG11 8NS UK

**Keywords:** Adipocyte, Adipose tissue, Inflammation, Mast cell

## Abstract

Excessive fatty acids and glucose uptake support the infiltration of adipose tissue (AT) by a variety of immune cells including neutrophils, pro-inflammatory M1 macrophages, and mast cells (MCs). These cells promote inflammation by releasing pro-inflammatory mediators. The involvement of MCs in AT biology is supported by their accumulation in the AT of obese individuals along with significantly higher serum levels of MC-derived tryptase. AT-resident MCs under the influence of locally derived adipokines such as leptin become activated and release pro-inflammatory cytokines including TNFα that worsens the inflammatory state. MCs support angiogenesis in AT by releasing chymase and inducing preadipocyte differentiation and also the proliferation of adipocytes through 15-deoxy-delta PGJ2/PPARγ interaction. Additionally, they contribute to the remodeling of the AT extracellular matrix (ECM) and play a role in the recruitment and activation of leukocytes. MC degranulation has been linked to brown adipocyte activation, and evidence indicates an important link between MCs and the appearance of BRITE/beige adipocytes in white AT. Cell crosstalk between MCs and AT-resident cells, mainly adipocytes and immune cells, shows that these cells play a critical role in the regulation of AT homeostasis and inflammation.

## Introduction

**Fig. 1:**
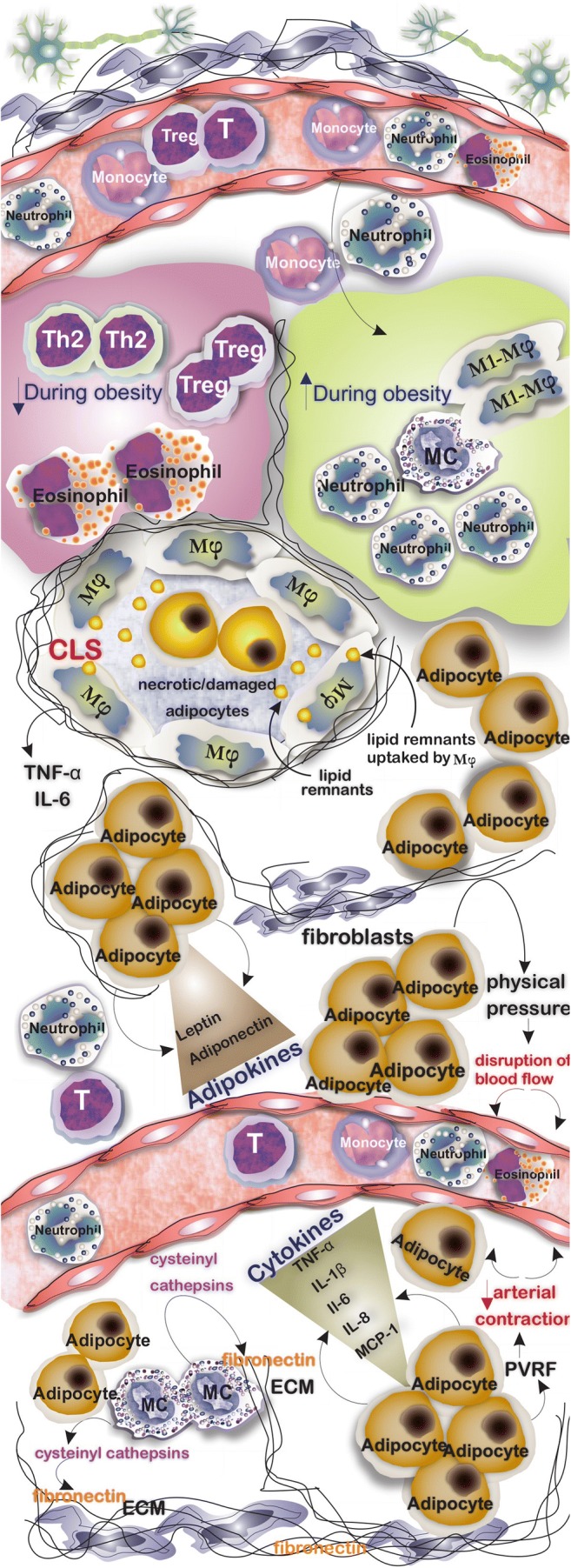
Adipocytes are the main cell population in AT. However, several types of cells are commonly found in AT which their number varies in lean and obese AT, for example, while the number of M1 macrophages, MCs, and neutrophils increases in obesity, the number of AT resident Th2, Treg, and eosinophils decreases. AT in obesity is infiltrated by inflammatory cells, and the formation of crown-like structures characterized by circled necrotic/damaged adipocytes with macrophages is a common finding. Adipocytes not only store lipids but also release several cytokines and adipokines that influence immune responses and hemostasis of the tissue. An increase in number and the size of adipocytes surrounding the vasculature system results in the formation of physical pressure and consequent disruption of blood flow. MCs through inducing the release of cysteinyl cathepsins from endothelial and adipocytes play a role in catabolizing fibronectin

Adipose tissue (AT) acts not only as an energy depot and regulator of energy homeostasis but also as an active endocrine organ capable of producing hormones and adipokines including leptin, adiponectin, TNF-α, IL-1β, IL-6, IL-8, and monocyte chemotactic protein-1 (MCP-1), [1, 2]. Obesity is accompanied by the accumulation of immune cells in AT after which they promote inflammation and negatively influence systemic metabolism [1]. Hyperplasia and hypertrophy of AT are two common findings during obesity through which AT expands in size [3]. Hypertrophy of adipocytes surrounded by a rigid extracellular matrix (ECM) causes physical pressure on the vasculature system that disturbs the blood flow of the tissue to promote inflammation and fibrosis [4]. Additionally, perivascular AT reduces arterial contraction by releasing perivascular-derived relaxation factors [5]. Alteration of AT residing cells during obesity has been well documented in which neutrophils, pro-inflammatory M1 macrophages, and mast cells (MCs) accumulate while populations including Th2, Treg, and eosinophils (populations that support anti-inflammatory responses and immunoregulation) are decreased [1] (Fig. 1). The bridging of inflammation and immunometabolism was highlighted by Hotamisligil et al. in 1993, by showing that TNF-α has elevated levels in obese fa/fa rats and its neutralization by a recombinant TNFR-IgG chimeric protein resulted in a marked increase in peripheral insulin-dependent uptake of glucose [6]. Investigations in humans then confirmed these findings. Elevated levels of TNF-α in obese individuals were shown to reduce during weight loss [7].

An increasing number of researchers have reported the accumulation of MCs in AT of obese individuals [8, 9]. MCs stimulate the release of cysteinyl cathepsins from vascular cells and adipocytes to catabolize ECM protein fibronectin to support adipogenesis [10]. Intraperitoneal injection of disodium cromoglycate (DSCG; a widely used inhibitor of MC activation and degranulation) of wild-type (WT) mice was reported to hamper the ability to gain body weight [11]. Interestingly, recent investigations linked the positive effects of traditional foods such as Chinese bitter melon and quercetin (a bioflavonoid found in dietary plants) with the capability of reducing body weight gain and insulin resistance (IR) to MC in which using such foods reduces the infiltration of MCs in AT and prevents the formation of an inflammatory microenvironment [12, 13]. Having a molecular understanding of the crosstalk between AT resident and infiltrated cells including monocytes and macrophages may shed light on better treatment of obesity and related diseases such as IR and diabetes.

## Mast Cell Origin, Development, and Function

**Fig. 2:**
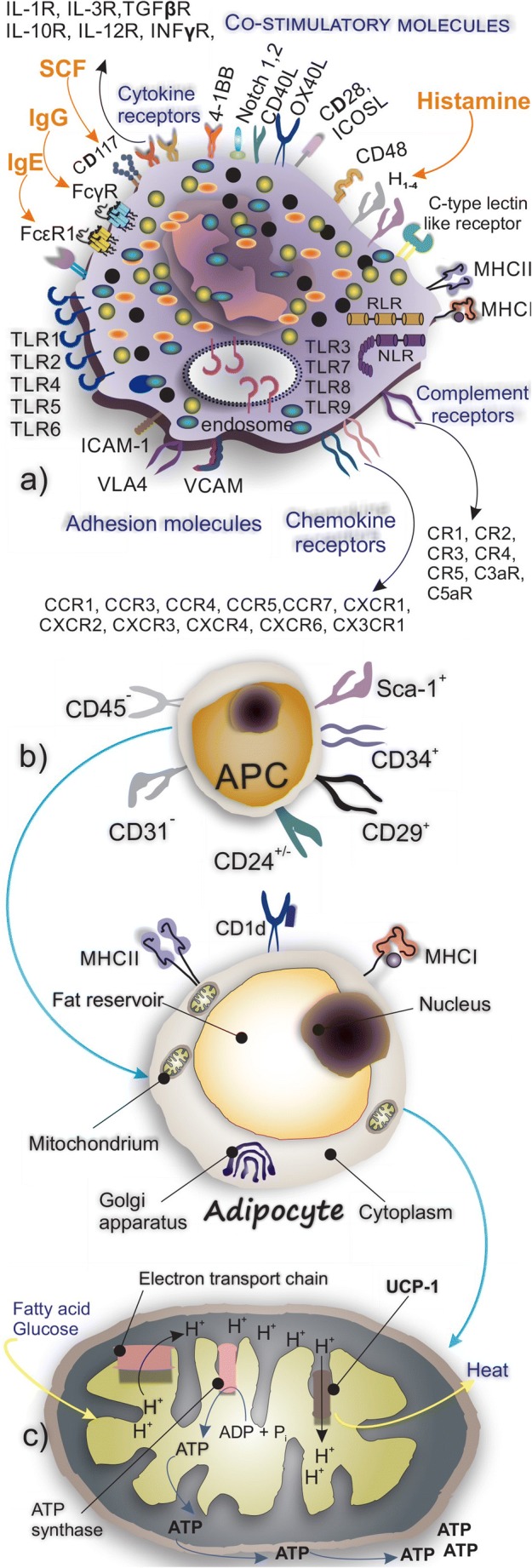
**a** MCs express a wide spectrum of receptors for chemokines and cytokines. Their main receptors for IgG, IgE, and SCF are depicted. **b** Adipocytes are derived from adipocyte progenitor cells. Their main surface receptors and molecules involved in the recognition of cells are shown. **c** Molecular mechanism of UCP1 in producing heat

MCs are cells of innate immunity that reside in tissues including AT and produce a range of pro-inflammatory cytokines [14]. They are granular long-lived cells that develop from CD34+/CD117+ pluripotent progenitor cells. These precursors, after being released from the bone marrow into the circulation, reach different target organs through chemokine and integrin-dependent trafficking [15, 16]. The progenitors under the influence of growth factors, mainly stem cell factor (SCF), differentiate and mature into functional MCs expressing FcεRI [17]. IgE-FcεRI interaction accounts for the main MC activation pathway through which MC degranulation occurs [17]. They produce and release three categories of molecules: (1) granule stored pre-formed mediators including histamine, heparin, tryptase, and chymase; (2) de novo synthesized mediators such as PAF, PDG2, and LTB4 and LTD4; and (3) cytokines including TNF-α, TGF-β, IL-1, IL-3, IL-5, IL-8, and IL-10 [18]. In humans, there are two subpopulations of MCs, namely MC_TC_, containing tryptase, chymase, carboxypeptidase, and cathepsin that can be found in connective tissues and MC_T_ which contain tryptase and are found mainly in the lung and gut [17] (Fig. 2a). MCs beyond their role in allergic reactions are involved in a variety of physiologic processes including angiogenesis (by releasing FGF, vascular endothelial growth factor (VEGF), and TGF-β) [19] and wound healing (through releasing IL-4, VEGF, and basic fibroblast growth factor (bFGF) [14]. Similar to other tissues, MCs reside in AT; however, their boosted infiltration into AT is a common finding during obesity. They promote the formation of an inflammatory milieu during obesity owing to their capability to release pro-inflammatory mediators [20, 21].

## Adipose Tissue Structure and Biology

Although AT was initially considered an inert storage organ of fat, this view changed over the past decades. It is now defined as a highly metabolic and active tissue, which reacts to certain chemicals and produces many adipokines (acting as an endocrine organ) that regulates metabolism [22]. AT is a loose connective tissue comprised of a variety of cells mainly adipocytes which are surrounded by a matrix of collagen fibers, fibroblasts, blood vessels, and immune cells [23]. Excess caloric intake is accompanied by fat deposition and growth of adipocytes followed by activation of endoplasmic reticulum stress and orchestration of oxidative stress responses [24]. Activation of these pathways results in the production and release of pro-inflammatory cytokines mainly IL-6 and TNF-α [24]. Formation of such a pro-inflammatory environment supports the activation of resident leukocytes and the infiltration of other inflammatory cells including macrophages, neutrophils, dendritic cells, lymphocytes, and MCs [24, 25]. The ECM plays a key role in homeostasis and regulation of AT. The accumulation of ECM proteins including collagen in the early stages of obesity contributes to tissue remodeling through which fibrosis and infiltration of pro-inflammatory leukocytes into the AT are promoted [26].

AT produces a wide range of adipokines that play key roles in the regulation of glucose and lipid metabolism [27], and their dysregulation has been linked to systemic inflammation [28]. (Table 1).

**Table 1:** List of AT-derived adipokines and their biologic functions

Adipokine	Immunobiologic function in AT	Ref
Leptin	Activates CD4 T cells and induce their production of TNF-α, IL-6, and IL-12 Activates MCs and induces the release of cysLTs Hypothalamic modulator of food intake, the regulator of energy expenditure Upregulates monocyte activation markers including CD11b, CD11c, MHC class II, CD25, CD38, and CD69 Promotes neutrophil chemoattraction and the production of ROS Leptin deficiency-induced obesity correlates with increased MCs in abdominal lymph nodes	[29] [30] [30] [29] [29] [31]
Adiponectin	The most abundant peptide secreted by adipocytes Acts as a regulator of thermogenesis Antagonizes TNF-α expression in adipocytes and macrophages Acts through AdipoR1 (mainly expressed in skeletal muscle) and AdipoR2 (predominantly expressed in the liver) Promote M2 macrophage polarization and improves insulin sensitivity	[23] [32] [29] [23] [33]
Lipocalin-2 (LCN2)	Also known as neutrophil gelatinase-associated lipocalin (NGAL) Upregulated in the presence of IFN-γ and TNF-α in obese individuals	[25] [29]
Retinol-binding protein 4 (RBP4)	Promotes IR and increases the T2D risk Majority of circulating RBP4 is found in complex with retinol RBP is a cardiometabolic marker in chronic pathologic conditions including MetS Activates APCs	[34] [35] [36] [36]
Fibroblast growth factor 21 (FGF21)	Regulates glucose and fat metabolism under fasting condition It is inactivated by fibroblast activation protein alpha (FAP-α) Engages its receptor FGFR1 and co-receptor β-Klotho Involved in fatty acid oxidation and lipid metabolism improves glucose tolerance	[37] [37] [38] [29]
Resistin	Produced mainly by macrophages and acts as an inflammatory molecule Secreted mainly by AT in rodents and macrophages in humans Regulates the production of TNFα and IL-6 in macrophages via activation of NF-κB signaling Binds to TLR4	[29] [39] [39] [39]
Visfatin	Also known as a pre-B cell colony-enhancing factor (PBEF), involved in chemoattraction of neutrophils Induces the production of cytokines in monocytes Acts through insulin receptor-1 and possesses hypoglycemic effect Activates monocytes, promotes the secretion of IL-1β, TNF-α, and IL-6	[28] [28] [27] [29]
Monocyte chemotactic protein-1 (MCP1)	Mediated the infiltration of monocyte and macrophage to the site of inflammation Its expression correlates with body BMI and adiposity	[40]
Fetuin-A	Promotes IR by inhibition of insulin receptor’s tyrosine kinase activity Mainly secreted by the liver and taken up by AT AT secreted fetuin-A increases in metabolic syndrome	[34] [41] [42]

AT of lean individuals produces and releases adipokines with anti-inflammatory properties mainly adiponectin and apelin while AT of obese individuals releases pro-inflammatory cytokines such as resistin, leptin, and visfatin [43]. Additionally, some investigations reveal the immunoregulatory properties of adipokines such as adiponectin that suppresses the activation of M1 macrophages while promoting the proliferation of the M2 subtype [44]. Proliferation and differentiation of preadipocytes or adipocyte progenitor cells within the stromal vascular fraction result in the formation of the new adipocytes [45]. Committed murine white adipocyte progenitors with CD31^−^, CD45^−^, CD29^+^, CD34^+^, Sca-1^+^, and CD24^+/−^ phenotype are involved in adipogenesis [45] (Fig. 2b).

Two types of AT, namely white adipose tissue (WAT) and brown adipose tissue (BAT), are known in human [23]. WAT is the main energy storage tissue, whereas, BAT dissipates energy in the form of heat and therefore plays a role in thermoregulation [46]. Both hypertrophy and hyperplasia of adipocytes are required for normal AT expansion. There is an approximately 8% rate of annual adipocyte turnover to match the rates of cell death [45]. Generally, white adipocytes act as the lipid storage units and release the stored free fatty acids during fasting periods while their counterparts brown adipocytes contribute to maintaining thermal homeostasis by burning glucose and lipids [47]. Brown adipocytes have a smaller size in comparison with white adipocytes, and their cytoplasm contains many smaller lipid droplets, a roundish nucleus and spherical mitochondria [47]. There are two distinct types of brown AT, the classical brown fat which is derived from a myf-5+ve cellular lineage and inducible brown fat that is generated in WAT from a non-myf-5 lineage [48]. Both types of brown adipocyte express uncoupling protein 1 (UCP1) on the inner mitochondrial membrane. The brown adipocytes present in WAT are termed BRITE (“brown in white”) or beige adipocytes [46]. Relatively few beige adipocytes are detected when animals are kept in normal vivarium conditions (22 °C). However, upon exposure to cold temperatures, the recruitment of beige adipocytes and also UCP1 increases [49]. The brown-like adipocytes in WAT depots are known for their high mitochondrial number and elevated expression of UCP1 [50] (Fig. 2c) and like classical brown fat, are able to respond to cyclic AMP [48]. Adipocytes express a variety of antigen-presenting molecules and complexes through which they mediate immune responses in other cell types, i.e., MHC I to mediate CD8 T cell responses, MHC II molecules for orchestration of CD4 T cell responses, and CD1d to present lipid antigens (including isoglobotrihexosylceramide, β-glucosylceramide, and plasmalogen lysophosphatidylethanolamine [51]) to iNKTs [52].

## Immune Cells Within the Adipose Tissue

### Cells of Innate Immunity

#### Role of Monocytes and Macrophage Within AT

A distinct feature of low-grade inflammation in AT is the formation of crown-like structures (CLS) which are syncytial arrangements comprised of encircled necrotic/damaged adipocytes with macrophages. The presence of CLS is associated with elevated levels of inflammatory mediators, mainly TNFα and prostaglandin E2 [53]. Investigations have revealed that these macrophages may resorb the lipid remnants of encircled dead adipocytes and also contribute to inflammation [54]. One difference between the two types of white AT is the lower number of CLS present in subcutaneous AT compared with visceral AT both in obese and lean mice [55]. The number of F4/80^+^CD11b^+^ macrophages increases in obese WAT. They produce IL-6, TNFα, and metalloproteinases (MMPs) which are associated with the development of IR and establishment of an inflammatory microenvironment [24, 56].

Classically activated macrophages (M1) with F4/80+ CD11b+ CD11c+ iNOS+ phenotype release high levels of pro-inflammatory cytokines including TNF-α, MCP-1, IL-1β, IL-6, IL-12, and iNOS, whereas alternatively activated macrophages (M2) having F4/80+CD11c-, CD301+, Arg1+, and CD206+ phenotype produce anti-inflammatory cytokines including IL-4, IL-10, and TGF-β1 [29]. The M2 population is normally predominant in AT of lean mice and a shift to M1 occurs as obesity progresses [29]. Activation of the M2 population contributes to the upregulation of immunomodulatory cells, mainly Tregs [57]. Macrophage-released CXCL2 which is upregulated in obesity stimulates the adhesion of neutrophils to WAT endothelial cells and may accelerate their infiltration in AT [58]. The M2 population plays a role in clearing and removal of non-functional adipocytes from AT and mediates the recruitment of adipocyte progenitors (APs) into AT. Clinical investigations showed that CD206+ M2-like macrophages crosstalk with APs through which they participate in adipogenesis, growth/differentiation of APs, and improve insulin sensitivity [59]. Arkan et al. found a macrophage association between inflammation and insulin resistance. They generated a mouse lacking IκB kinase β in myeloid cells including macrophages and reported that these mice have higher insulin sensitivity, suggesting that inhibition of IKK-β may be promising in the treatment of IR [60]. Very low-density lipoprotein receptor signaling in macrophages mediates pro-inflammatory responses and supports the polarization of the M1-like phenotype, and during obesity, expression of this receptor is increased [61]. These findings support that macrophages induce inflammation in AT [61]. The recruitment of monocytes in AT is facilitated by MCP-1/CCR2 interaction [33]. Dendritic cells (CD11c^+^CD1c^+^ in human and CD11c^high^F4/80^low^ in mouse) have been reported to accumulate in AT during obesity and act in favor of differentiation of Th17 cells [62]. Eosinophils play a role in metabolic homeostasis by supporting the presence of M2 macrophages through releasing IL-4 and IL-13 [63]. The mechanism of action may include engaging PPARγ receptors expressed on M2 macrophages by eosinophil-derived IL-4 and IL-13 [63].

#### Role of Neutrophils Within AT

Neutrophils are among the first cells that infiltrate AT upon starting a high-fat diet (HFD) in mice [64]. They can be attracted to AT by IL-8 secreted from adipocytes and CXCL2 secreted by macrophages [24, 58]. Neutrophil-secreted elastase contributes to the polarization of M1 macrophages via TLR-4 and degrades insulin receptor substrate-1 which leads to decreased insulin sensitivity of the AT [24]. Within the AT, neutrophils release pro-inflammatory cytokines including IL-8, CCL2, MMP-9, and myeloperoxidase which aggravate the inflammation state [58]. Evidence for the role neutrophil activation in obesity includes the increased expression of the activation marker CD66b and increased circulatory neutrophil-released myeloperoxidase and calprotectin. Myeloperoxidase contributes to the development of obesity and its ablation or inhibition prevents weight gain and IR [65]. Additionally, neutrophil-released superoxides induce apoptosis and activate macrophages through which they contribute to the formation of a pro-inflammatory state [29]. Elastase among neutrophil-released mediators is of importance in inducing IR, and inhibition of elastase improves insulin sensitivity [64].

### Cells of Adaptive Immunity

**Fig. 3:**
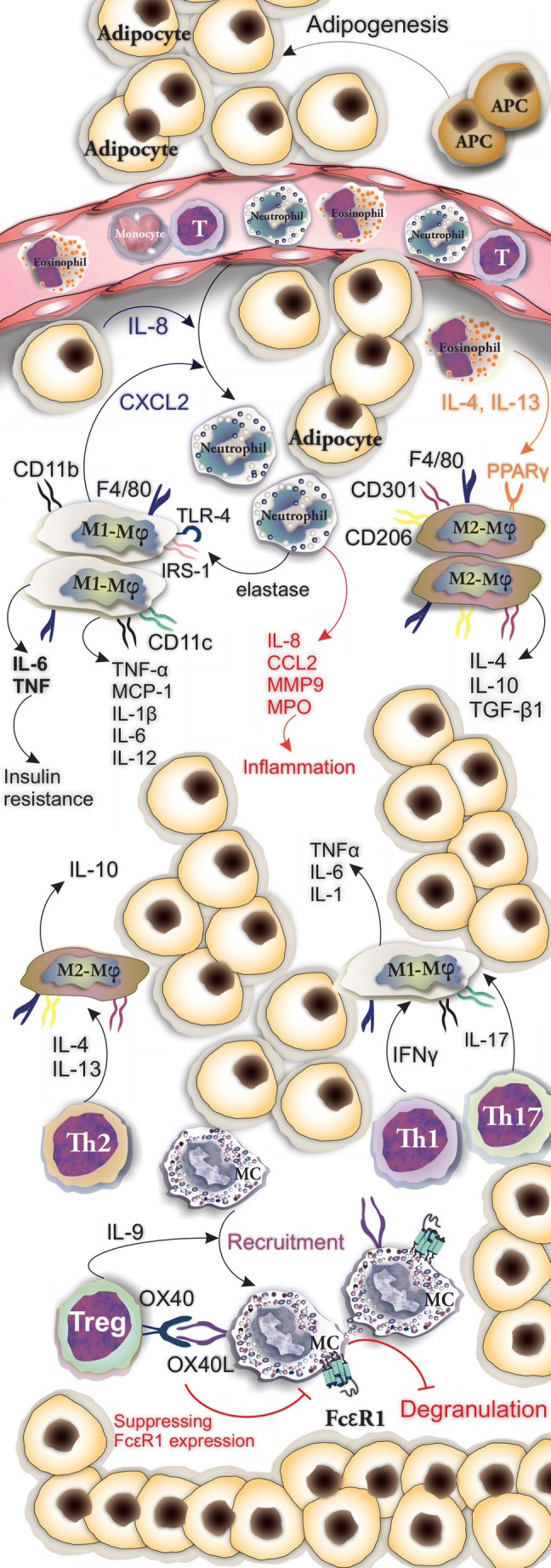
Involvement of cells of innate and adaptive immunity in the orchestration of responses in AT. Inflammatory and anti-inflammatory activity of M1 and M2 macrophages are shown

Like the cells of innate immunity, orchestration of immune responses by cells of adaptive immunity determines the metabolism and biology of AT. IR is associated with increases in cell populations with a pro-inflammatory phenotype including Th1, Th17, CD8+ cytotoxic T cells, and B-2 over those cell populations with regulatory properties mainly Treg and B-1a [66]. IFNγ and IL-17 secreted by Th1 and Th17 cells, respectively activate the pro-inflammatory functions of macrophages through the release of TNF-α, IL-6, and IL-1. In contrast, IL-4 and IL-13 secreted by Th2 cells induce macrophage differentiation into the anti-inflammatory IL-10 secreting M2 subset [67]. Tregs are the predominant T cell population in AT of lean mice; however, under HFD, their number decreases, whereas Th1 cells increase [62]. Interaction between OX40 on Treg and OX40L on MCs results in suppression of MC degranulation and FcεRI expression. IL-9 produced by Tregs plays a role in the recruitment of MCs in AT [68] (Fig. 3). Feuerer et al. investigated the role of Tregs in AT and IR by inducing selective apoptosis in Tregs. For this purpose, they used diphtheria toxin receptor (DTR) expressing mice in which the DTR expression was under the control of the Foxp3 promoter. Following Tregs depletion in these mice by diphtheria toxin administration, IL-6, TNF, and RANTES expression in fat increased. They also reported elevated levels of insulin in Treg-depleted mice which could be a sign of IR [56].

## Adipose Tissue Residing Mast Cells

Immunostaining of AT sections for tryptase and CD117 is a common approach used to determine the presence of MC populations within AT [11]. MCs with their pro-inflammatory profile of mediators promote the state of inflammation and participate in apoptosis and angiogenesis and may contribute to the progression of obesity and glucose intolerance via the release of IL-6 and IFN-γ [11] (Table 2).

**Table 2:** Bio-function of MC mediators in adipose tissue biology

MC mediator	Bio-function in AT	Ref
Chymase	Promotes angiogenesis in AT	[24]
IFN-γ	Activation of AT-resident macrophages	[24]
MMP-9	Activation of AT-resident macrophages	[24]
Tryptase	Activates PAR2 through which upregulates the expression of inflammatory factors, such as TNF-α, IL-1β, and IL-6 in endothelial cells	[2]
MCP-6	Promotes the fibrosis in AT	[69]
IL-6	Induces the inflammation in AT	[8]
MCP-1	Induces the inflammation in AT	[8]
TNF-α	Pro-inflammatory cytokine involved in the pathogenesis of obesity, i.e., IR	[31]

The MC population in adipose tissue is dynamic in nature showing changes associated with tissue remodeling in obesity. Both maturation and differentiation of MCs could occur in WAT as c-Kit^+^Thy^−^1^lo^Lin^−^Sca^+^ cells found in mouse subcutaneous fat pads differentiate into MMCs in vitro [22, 68]. Based on the anatomical positions of fat pads such as subcutaneous and epididymal fat, MCs show different activity and distribution [31]. For instance, visceral WAT of obese mice shows higher numbers of MCs compared with those of lean mice. Moreover, there is no significant difference in MC number in subcutaneous WAT between obese and lean mice [68]. Profound differences have been found when comparing MCs in the adipose tissue at morbid obesity and after bariatric surgery-induced weight loss. Surprisingly, there was a dramatic increase in the adipose resident MCs in the weight loss group with a ten-fold increase in the visceral and four-fold increase in subcutaneous adipose tissue [70]. MC-deficient mice and MC-stabilizing agents such as disodium cromoglycate have served to attempt to define the roles of AT-resident MCs in obesity and IR. Additionally, pharmaceutical reduction of MCs has been reported when pioglitazone, a PPARγ agonist, was applied [4, 11].

## MC Crosstalk with Cells of Adipose Tissue

During obesity, MCs accumulate in AT where they are distributed among adipocytes or around vessels. The contribution of MCs to promote fibrosis has been investigated and their presence in fibrosis bundles and the proximity of fibrosis surrounding vessels has been reported [8]. Within AT, MCs secrete mediators that influence the immune responses of surrounding immune and non-immune cells. MC-released IFN-γ, chymase, tryptase, IL-6, and cysteinyl cathepsins are capable of activating vascular cells and adipocytes through which they support angiogenesis and differentiation of adipocytes [71]. Adipocytes release adipocytokines that may induce a series of immune responses in surrounding cells within AT. For instance, leptin acts on the leptin receptor expressed by MCs and triggers the release of mediators including cysLTs and CCL3 [30, 72]. MC mediators including IFN-γ, MMP-9, and phospholipase A2 regulate activation of macrophages [24]. MC-derived MCP-6 has been reported to induce collagen 5 expression in AT-resident fibroblasts and plays a role in fibrosis [69]. Prostaglandins are mediators produced by MCs and the metabolite, 15-deoxy-delta-12,14-PGJ2 (15-deoxy-delta PGJ2), acts as the endogenous ligand of PPARγ [73]. Tanaka et al. showed that supernatants obtained from MCs activated by calcium ionophore contained 15-deoxy-delta PGJ2 which induces adipogenesis of 3T3-L1 cells and primary preadipocytes [73].

## Function of MC Mediators in Adipose Tissue—Lessons from Animal Models

**Fig. 4:**
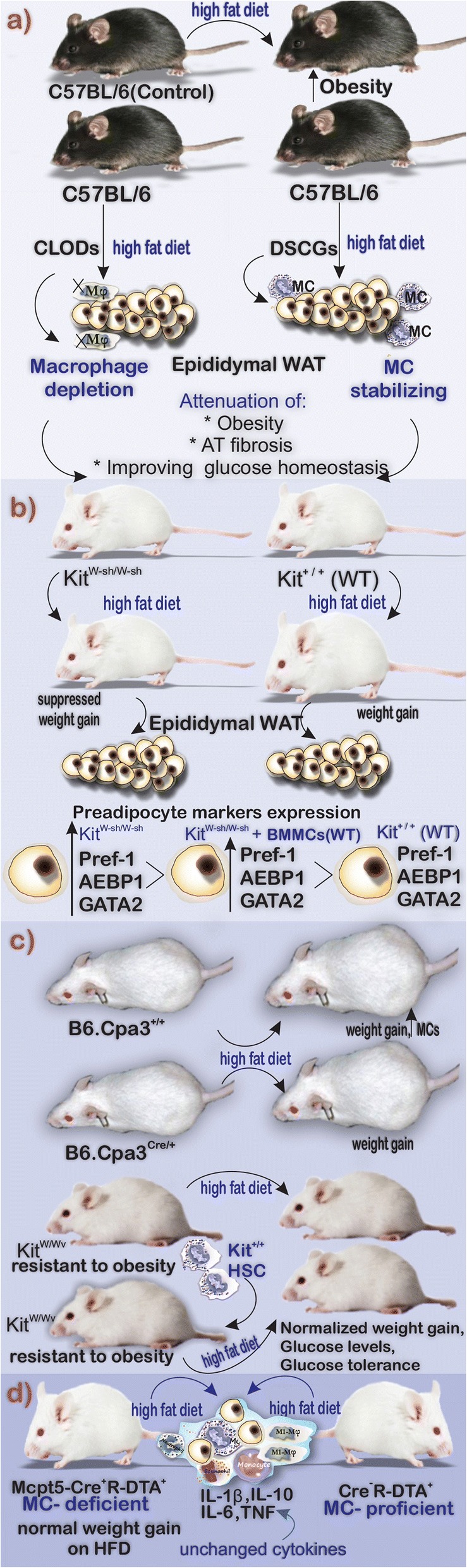
Graphic summary of three animal models to show the involvement of MCs in diet-induced obesity. (WT: wild type, AT: adipose tissue, HFD: high-fat diet, HSC:hematopoietic stem cell, CLODs: clodronate sodium liposomes, DSCGs: disodium cromoglycate sodium liposomes, BMMCs: Bone marrow-derived mast cells)

The observed increase in MCs in adipose tissue in obesity led to the study of their role in metabolic dysregulation associated with inflammation. Several different in vivo models have resulted in a degree of controversy with profoundly different phenotypes observed when different approaches have been taken to investigate the roles of AT-resident MCs. The involvement of MCs in obesity and IR has been investigated by the in vivo application of MC stabilizers that block degranulation and the release of mediators. Kumar et al. put C57BL/6 mice on HFD to initiate a progressive glucose intolerance, IR, and AT senescence [74]. Their flow cytometric results showed an interesting fluctuation in AT cellularity during the HFD diet. M1 macrophages showed a rise from nearly 1.4% of total immune cells and reached 15.7 ± 1.5% at 20 weeks. Eosinophils, the presence of which positively correlates to insulin sensitivity showed a decrease from 8.7 ± 1.04% at the early phase of 4 weeks to 5.6 ± 0.6% at 16 weeks and their population restored at 20 weeks. FcɛRIa^+^ MCs showed a fluctuation in which their population rose from 39.5 ± 2.8% at 4 weeks and dropped in number to 27 ± 1% at 12 weeks and then reached to 32.62 ± 1.5% at 20 weeks of HFD. To investigate the role of macrophages, they were depleted using clodronate sodium liposomes (CLODs). Additionally, MCs were stabilized by disodium cromoglycate sodium liposomes (DSCGs). The strategy indicated that macrophages and MCs are involved in the progression of obesity, AT fibrosis, and glucose homeostasis [74]. A notable rise in serum glycerol level in both CLOD- and DSCG-treated mice showed the mobilization and burning of fat [74] (Fig. 4a).

Initial studies of the role of AT MCs under physiological conditions were investigated by Ishijima et al. by assessing the MC-deficient Kit^W-sh/W-sh^ mice [75]. These mice, due to the presence of the W-sash (W(sh)) inversion mutation in their white spotting (W) locus, lack the normal signaling of c-kit tyrosine kinase when compared with wild-type Kit^+/+^ mice [76]. Kit^W-sh/W-sh^ mice are fertile and non-anemic but histologically lack a variety of cells mainly MCs, melanocytes, and interstitial cells of Cajal [77]. Body weight gain induced by HFD was suppressed in the Kit^W-sh/W-sh^ mice compared with the control group. Investigations of the levels of the preadipocyte markers Pref-1, AEBP1, and GATA2 revealed a notably higher expression in the epididymal WAT and stromal vascular fraction of the MC-deficient mice comparing with counterpart WT mice [75]. They suggested that MCs have positive effects on the transition of preadipocytes to mature adipocytes [75] (Fig. 4b). Liu et al. to provide a line of evidence on MC involvement in obesity investigated the effects of a 12-week Western diet. They concluded that Kit^W-sh/W-sh^ mice gained less weight in comparison with WT counterparts. Moreover, using intraperitoneal (i.p.) injections of DSCG, they reported the positive effects of MC stabilizer to reduce the weight gain in mice [11]. Additionally, this group investigated the role of MC mediators in obesity progression. They reconstituted Kit^W-sh/W-sh^ mice with bone marrow mononuclear cells (BMMCs) prepared in vitro from WT mice and mice lacking MC cytokines IL-6 (Il6^−/−^), TNF-α (Tnf^−/−^), and IFN-γ (Ifng^−/−^) and put them on Western diet for 13 weeks. They reported that Kit^W-sh/W-sh^ mice reconstituted with WT and Tnf^−/−^ gained more weight when compared with non-reconstituted mice. Interestingly, Kit^W-sh/W-sh^ mice that received WT and Tnf^−/−^ BMMCs were found with higher serum glucose levels, leptin, and insulin. They also reported that Kit^W-sh/W-sh^ mice reconstituted with Il6^*−/−*^ and Ifng^−/−^ BMMCs had improved glucose tolerance [11].

Although the initial studies using genetic mouse models with *c-kit* mutation indicated the involvement of MCs in obesity, several investigators have reported results that are inconsistent with these findings following the application of alternative genetic models. Gutierrez et al. found that Kit deficiency and not the lack of MCs play a central role in metabolic improvements when exposed to HFD. They highlighted the role of Kit deficiency to protect the mice from HFD-induced obesity which was due to the hematopoietic system. This group of researchers studied the role of MCs in obesity in two MC-deficient mice models, Kit^W/Wv^ (mice with deficiency in Kit) and Cpa3^Cre/+^ (mice with Kit-independent MC deficiency), and studied the process of obesity and IR after employing diet-induced obesity [78]. They first fed Cpa3^Cre/+^ and Cpa3^+/+^ (as control) with HFD and low-fat diet for 16 weeks and reported identical weight gain in each group. Investigation of AT of these mice showed that obese Cpa3^+/+^ mice had a higher number of MCs when compared with the lean Cpa3^+/+^ mice. Interestingly, the reconstitution of Kit^W/Wv^ mice with Cpa3^Cre/+^ bone marrow could completely normalize stem and progenitor cell compartments. Additionally, Kit^+/+^ hematopoietic transplantation could reverse all the metabolic phenotypes of the Kit^W/Wv^ mice including weight gain during HFD, baseline hyperglycemia, and loss of protection from glucose tolerance [78] (Fig. 4c). An additional model of MC deficiency has utilized a mouse line in which the Cre-recombinase-dependent expression of diphtheria toxin is triggered in cells under the control of the mast cell protease (Mcpt) 5 promoter. Mcpt5-Cre^+^R-DTA^+^ and Cre-negative R-DTA^+^ mice were subjected to HFD for 21 weeks. No difference in terms of accumulation of M1-macrophages, or upregulation of inflammatory cytokines including IL-1β, IL-6, IL-10, and TNF, was reported. Furthermore, MC deficiency had no marked differences in obesity and obesity-related dysregulation [79]. Although MC numbers increase upon exposure to high-fat diet, the weight of evidence, taking into account the different genetic models used, indicates that the absence of these cells does not protect from obesity and IR. There are lines of evidence linking AT residing MCs to other pathologic conditions. For example, periaortic perivascular adipose tissue of patients with abdominal aortic aneurysm was shown to be populated by leukocytes including MCs. The presence and capability of MCs to produce pro-inflammatory mediators could aggravate the condition [80].

## Role of MC in AT Browning

There is accumulating evidence supporting a role for MCs in the browning of white adipocytes. It has been found that repeated cold exposure promotes beiging of human subcutaneous WAT and it is associated with increases in adipose tissue MC recruitment [9]. Recently, Finlin et al. reported that MCs release histamine in response to cold, and this mediator induces the expression of UCP1 that is capable of uncoupling mitochondrial oxidative respiration and generating heat [81]. Such a mechanism may hamper the process of obesity by increasing energy expenditure. A study of seasonal beiging of human subcutaneous WAT identified a set of immune markers that were predictive of the *UCP1* gene expression. There was a correlation with IL-4 and carboxypeptidase-A3 (CPA3), a protease that is specifically expressed by MCs [81]. As IL-4 expression was also correlated with CPA3, it could indicate that MCs may be a source of this cytokine. Importantly, in vitro studies found that MC degranulation and histamine release promoted UCP1 expression and stimulated lipolysis. Furthermore, histamine treatment of adipocytes potently induced UCP1 protein and mRNA along with histamine receptors. The primary mechanism of brown and beige adipocyte activation is via the sympathetic nervous system through norepinephrine action. Importantly, it has also been reported that MCs express β-adrenergic receptors and can respond to norepinephrine to degranulate and release histamine [9]. BAT is highly vascularized with a complex network of blood vessels, and when activated, there is an increase in blood flow [82]. In the rat, expression of histamine H3 receptors has been found in capillaries within BAT. This raises the possibility that histamine signaling could be involved in the regulation of thermogenesis by acting as a vasodilator on the endothelial cells [83].

A recent report has indicated that rather than having a positive effect on browning of WAT, MCs have an inhibitory role in this process. Zhang et al. studied the process of AT browning in Kit^w-sh/w-sh^ and MC-stabilized (WT) models which received a chow diet. They reported that MC inactivation induces the proliferation of adipocyte precursors with platelet-derived growth factor receptor A (PDGFRα) expression, supports the beige adipocyte differentiation, and improves the thermogenesis in subcutaneous AT. Gene expression analysis showed upregulation of key brown fat genes in the subcutaneous AT of Kit^w-sh/w-sh^ mice compared with WT controls including *Ucp1*, *Cidea*, and *Elovl3*. Immunostaining of UCP1 of subcutaneous AT samples obtained from Kit^w-sh/w-sh^ mice receiving DSCG showed that they have a higher number of UCP1^+^ beige cells compared with WT mice. Moreover, considering the role of serotonin in energy balance and AT browning and that serotonin suppresses the expression of UCP1 [84], Zhang and colleagues investigated tryptophan hydroxylase 1 (TPH1) which catalyzes the production of serotonin from tryptophan in Kit^w-sh/w-sh^ mice and DSCG-treated WT mice. They reported a significant suppression of the enzyme in these two models in comparison with the control group. Further investigation using WT model receiving TPH1 inhibitor (LX1031) could support their findings by showing that TPH1 inhibition increases the UCP1 expression and that serotonin is capable of inhibition of browning in subcutaneous AT [84]. The data from this study indicate that there is a profound browning of the WAT in the Kit^w-sh/w-sh^ mouse and this could help explain the obesity resistance of the model. However, an examination of the available microarray data on the expression of genes in AT of low-fat diet-fed Kit^W/Wv^, Cpa3^Cre/+^, and Cpa3^+/+^ (GEO: GSE67091, and analyzed using GEO2R [78]) did not reveal any differences in the levels of UCP1 or Cidea. To definitively conclude that browning is associated with loss of MCs within the AT will require further investigations using alternative genetic models that are not dependent on c-kit mutations. There is a wide range of evidence supporting the role of MCs in the browning of adipose tissue. However, further research is required to fully understand the actions of MCs in white AT and MC-derived histamine in BAT and beige fat activation.

## Discussion and Conclusion

MCs in addition to orchestrating the inflammatory responses in AT during the progression of obesity influence adipocyte reaction to physical changes such as beiging in response to cold to promote the thermogenesis [9]. The molecular mechanisms by which MCs respond to environmental physical changes have not been completely understood. In addition to a heterogeneous population of AT-resident cells, their interplay and similarity in expression of several receptors make AT immunobiology much more complicated. In this regard, expression of PAR2 (a G protein-coupled receptor which acts as a receptor for MC-released tryptase [14]) by not only adipocytes but also other AT-resident cells, including macrophages, makes the role of this receptor in AT biology in response to MC-released tryptase even more complex. Interestingly, even the expression levels of PAR2 vary among different strains of mice which are widely used in AT biology-related investigations. For example, ob/ob mice have significantly higher levels of PAR2 receptors in comparison with C57BL/6J (C57) mice [2]. Overexpression of PAR2 in AT during obesity and the possibility of blocking it by antagonists makes it a potential biomarker and pharmaceutical target in controlling obesity. In this regard, Lim et al. used GB88, a novel PAR2 antagonist in rats, and reported its benefits in attenuation of adiposity, AT inflammation, and reducing infiltration of macrophages and MCs [85]. Further investigation is needed to reveal the complex interaction of MCs and other AT-resident cells.

Recent studies aimed to clarify that the MC-adipocyte interactions have provided promising results in MC biology. In this regard, Paupert et al. developed a method to generate pure and functional human MCs in 3 weeks from AT. They cultured the stromal vascular fraction of AT as spheroids in serum-free medium enriched with SCF. Obtained human MCs were able to degranulate in the presence of IgE, C5a, substance P, and compound 48/80 and could produce prostaglandins, TNF-α, IL-6, GM-CSF, chymase, tryptase, and CPA3. These AT-derived MCs had the advantages of available MC lines due to expressing FCεRI (unlike HMC-1 cells) or responding to SCF (unlike LUVA cells) [86]. MC ablation or stabilization due to the reported results may be promising strategies to control obesity and IR. There is compelling evidence implying the promising effects of using MC stabilizers in controlling obesity and induced diabetes in rodent models. However, only a small number of papers have reported such investigations in humans. In this regard, El-Haggar et al. studied ketotifen (a common MC stabilizer) in obese patients with T2D treated with glimepiride. They concluded that co-administration of ketotifen twice daily with glimepiride alleviates glycemic and inflammatory processes in treated obese individuals with T2D [87]. Additionally, the exact role of MCs in the pathology of metabolic syndrome (MetS) needs to be investigated. Most recently, Gurung et al. provided a line of evidence that subcutaneous adipose tissue (SAT) residing MCs of individuals with MetS may contribute to insulin resistance. Their results showed that the numbers of MCs (1) increase in SAT of the studied individuals and (2) positively correlate with IR in AT and the levels of glucose [88].

Although an overview of findings indicates that the absence of MCs does not prevent obesity, investigations aimed to reveal the interactions of MCs, and adipocytes show that MCs accumulate in AT of obese individuals including both mouse models and humans. Moreover, AT-resident MCs under the influence of AT-derived cytokines become activated and release pro-inflammatory cytokines that worsen the inflammatory state. Besides, MCs play a role in the remodeling of AT ECM and contribute to the recruitment of leukocytes with inflammatory activity. Further investigations are required to fully define the crosstalk between MCs and other AT-resident cells and how this affects inflammation, energy homeostasis, and induction of beige adipocytes.

## Abbreviations

AT: Adipose tissue
BMMC: Bone marrow-derived mast cells
HFD: High-fat diet
IR: Insulin resistance
MC: Mast cell
MCP-1: Monocyte chemoattractant protein-1
PPARγ: Peroxisome proliferator-activated receptor-gamma
T2D: Type 2 diabetes
WAT: White adipose tissue
VEGF: Vascular endothelial growth factor
bFGF: Basic fibroblast growth factor
